# Neuroanatomical Correlates of Mild-to-Moderate Depression: Memory Ability Mediates the Association Between Gray Matter Volume and Antidepressant Treatment Outcome

**DOI:** 10.3389/fnins.2022.872228

**Published:** 2022-03-30

**Authors:** Hong Li, Junjie Wang, Sha Liu, Zhifen Liu, Yong Xu

**Affiliations:** ^1^Shanxi Key Laboratory of Artificial Intelligence Assisted Diagnosis and Treatment for Mental Disorder, First Hospital of Shanxi Medical University, Taiyuan, China; ^2^Department of Psychiatry, First Hospital of Shanxi Medical University, Taiyuan, China; ^3^Department of Mental Health, Shanxi Medical University, Taiyuan, China

**Keywords:** mild to moderate depression (MMD), magnetic resonance imaging (MRI), right amygdala, memory ability, Shugan Jieyu capsule

## Abstract

Mild-to-moderate depression (MMD) is frequently encountered in clinical practice. Investigating the brain mechanism and its relationship with symptoms in patients with MMD can help us understand the occurrence and development of depression, thus optimizing the prevention and treatment of depression. Shugan Jieyu capsule (SG), a traditional Chinese medicine, is commonly used to ameliorate emotional and cognitive symptoms induced by patients with MMD. Combining clinical assessments and magnetic resonance imaging (MRI), we obtained the emotional and cognitive status of MMD patients and also explored the structural and functional alterations in MMD patients after SG treatments. Structural MRI demonstrated that the gray matter volumes of the left thalamus, right thalamus, and right amygdala in MMD patients were significantly smaller than in healthy controls, and the right amygdala volume was negatively related to depression symptoms in MMD patients. Resting-state functional MRI data demonstrated that MMD patients exhibited decreased temporal coupling between the right amygdala and nucleus accumbens, which was further associated with the severity of depression. Furthermore, right amygdala volume at baseline served as a significant predictor to identify the treatment outcome after 8 weeks of SG treatment in the patients’ group, and importantly, the memory ability mediated the relationship from right amygdala volume to the treatment outcome. These data revealed the structural and functional deficits in the right amygdala, which were highly correlated with the symptoms of depression and its cognitive ability, likely predicting treatment outcome. Therefore, this study strengthened our understanding of the pathogenesis of MMD, which is hoped that it will contribute to tailoring a personalized method for treating the patients.

## Introduction

Depression is a debilitating mental illness comorbid with a series of emotional, cognitive, and behavioral symptoms, affecting an estimated 264 million people worldwide (GBD 2017 Disease and Injury Incidence and Prevalence Collaborators, 2018). It is reported that there are more than 95 million people with depression in China, with a lifetime prevalence of depression of 6.9% and a 12-month prevalence of 3.6% (Huang et al., 2019). According to the severity of symptoms, depression can be clinically classified as mild, moderate, or severe. Mild to moderate depression (MMD), including less than five symptoms of depression (Liu et al., 2020), occurs more frequently than severe depression in clinical practice (Meng et al., 2021). Given its high incidence, it is important to explore the cognitive and neurobiological factors that relate to the onset, maintenance, and recurrence of MMD. Technologically, neuroimaging technologies can help us to identify objective neurobiological features reflecting underlying pathophysiologic processes in a given psychiatric illness, which can ultimately elaborate the development of personalized treatments based on a better understanding of these potential processes (Phillips et al., 2015).

In recent years, the in-depth application of the magnetic resonance imaging (MRI) technique in clinical research has greatly promoted the understanding of brain mechanisms related to depression. A large body of animal and human neuroimaging studies have demonstrated both structural and functional abnormalities of prefrontal-limbic brain regions, including the prefrontal cortex, amygdala, insula, and anterior cingulate cortex in depression (Kim et al., 2012; Whittle et al., 2014). A key role in depression pathogenesis seems to be played by the amygdala, which is known to be involved in emotional processing, fear response, and cognitive execution (Delgado et al., 2008; Etkin et al., 2009; Ressler, 2010; Comte et al., 2016). Indeed, one of the brain functional abnormalities in depression is serotonergically regulated implicit emotion adjustment neural circuitry centered on the amygdala and different medial prefrontal cortical regions (Mayberg et al., 2000; Kumar et al., 2008; Pizzagalli, 2011). However, the amygdala-related neural mechanisms underlying MMD patients remain unclear (von Gunten et al., 2000; Weniger et al., 2006). More importantly, it is unclear how early neuroimaging changes in depression-related brain regions, such as the amygdala, moderated or mediated subsequent treatment response.

From the angle of symptomatology, studies on mental disorders have focused on emotional symptoms traditionally. However, the cognitive deficit of improper control is also prominent, which seriously affects the quality of life (Millan et al., 2012). MMD occurs primarily in the form of emotional and cognitive symptoms, in which cognitive impairment may hinder functional recovery. It is worth noting that cognitive remission is gradually recognized as a novel objective for the treatment of MMD (Bortolato et al., 2016). Cognitive dysfunction, especially memory dysfunction, is a frequent residual manifestation in MMD and may persist during the remitted phase. In fact, cognitive impairment is considered to be an important feature that seriously affects the recovery of function after antidepressant treatment (Zuckerman et al., 2018). Based on this, cognitive ability is considered to play a potential mediating role in the antidepressant treatment of MMD patients. However, there is a lack of relevant research to support this hypothesis.

Shugan Jieyu capsule (SG), a Chinese herbal medicine, is mainly composed of *Eleutherococcus senticosus* (also known as *Acanthopanax senticosus*) (Eleuthero, 2006) and *Hypericum perforatum* [also known as St John’s Wort (2006)] (the proportion is approximately 5:6). Both *E. senticosus* and *H. perforatum* have been demonstrated to be effective in treating depression and cognition impairment (Sithisarn et al., 2013; Ben-Eliezer and Yechiam, 2016). SG has been licensed in China since 2008 and is widely used to treat depression (Sun et al., 2009). The effect of SG in ameliorating depressive symptoms is similar to that of escitalopram, but due to its fewer adverse reactions in relieving the symptoms of depression, it has been recommended for the individualized treatment of MMD (Zhang et al., 2014; Sun et al., 2018; Huang and Kuang, 2019).

In the current study, we adopted MRI and psychometric techniques to detect the treatment efficacy of SG and its underlying neural mechanisms in patients with MMD. Based on previous studies, we hypothesized that (1) the efficacy of SG in the treatment of MMD might be reflected in the structural and functional changes of brain regions related to emotion and cognition, especially the amygdala, and (2) the efficacy of SG in the treatment of MMD might be affected by the patient’s cognitive ability, especially memory ability. The results of this study may add new insights based on traditional Chinese medicine intervention into the understanding of the neurobiological mechanisms implicated for the amygdala and its relationships with emotional symptoms as well as memory impairments at an initial developmental period of depression.

## Materials and Methods

### Participants

In this study, patients were recruited from the Department of Psychiatry, the First Hospital of Shanxi Medical University, and met the following criteria: (1) Major depression disorder (MDD) diagnosis with an actual mild-to-moderate episode according to the *Diagnostic and Statistical Manual of Mental Disorders, Fifth Edition* criteria; (2) Score 7 < 24-item Hamilton Depression Rating Scale (HAMD-24) scores <24; (3) 18 years ≤ age ≤ 50 years; (4) right-handedness; (5) no comorbid axis I diagnosis that is all psychological diagnostic categories; (6) no history of neurological diseases; and (7) no physical limitations prohibiting them from undergoing an MRI examination. Two patients were excluded because of incomplete clinical evaluation. In addition, all healthy controls were recruited from the community nearby. The criteria for healthy controls included: (1) HAMD-24 scores < 7; (2) no family history of psychosis; and (3) no psychological problems and symptoms of psychopathology.

Finally, 47 right-handed MMD patients (17 men; mean age 26.12 ± 5.06 years) and 43 age- and sex-matched right-handed healthy controls (13 men; mean age 26.27 ± 5.69 years) participated in the study. This study was conducted on the basis of the latest version of the Declaration of Helsinki Declaration of 1975, as revised in 2008. The Ethics Committee of Shanxi Medical University approved this study. Participants included in this study all signed informed consent forms.

### Clinical Assessments

Depressive and anxiety symptoms were assessed based on the HAMD-24 items and Hamilton Anxiety Rating Scale (HAMA)—14 items by two research psychiatrists independently (Bodescu et al., 2015). Both scales have been confirmed to have good reliability coefficients. In addition, patients with MMD and healthy controls also underwent neurocognitive assessment *via* Wechsler Memory Scale (WMS), which has presented good reliability and validity (Bodescu et al., 2015). The WMS includes 10 subtests, particularly questions that would determine the participants’ long-term memory, short-term memory, and transient memory. For MMD patients, Pearson’s correlation analyses between the amygdala volumes, Wechsler memory, and severity in depression and anxiety symptoms were performed at baseline while controlling for nuisance variables, including age, sex, and years of education.

### Medication Treatment

After pretests, MMD patients included were assigned to take a Chinese herbal medicine named SG, which is mainly made up of *Acanthopanax* and *H. perforatum* (Yang, 2015), licensed and widely used in China since 2008 (Sun et al., 2009). According to the recommended medication dose, MMD patients took 1.44 g of SG per day, including 0.72 g in the morning and 0.72 g in the evening for 8 weeks. During the treatment, all participants were acquired to complete the HAMD assessment for two time points (4 and 8 weeks).

### Clinical Outcomes

Clinical outcome was defined by the rating of the HAMD scales. Remission was scaled as a HAMD score < = 7, and treatment failure was scaled as HAMD score > 7 at both weeks 4 and 8.

### Magnetic Resonance Imaging Acquisition

All participants lay supine on the bed of a Siemens Skyra 3.0-T MRI scanner with a standard 32-channel head coil to collect the MRI data at the Shanxi Provincial People’s Hospital. T1-weighted anatomical data were acquired by covering the entire brain using the MPRAGE sequence (repetition time/echo time = 2,300/2.95 ms, FA = 9°, data matrix = 225 × 240, 160 slices, slice thickness = 1.2 mm). Functional image was obtained using an EPI sequence (repetition time/echo time = 2,500/3.0 ms, FA = 90°, field of view = 240 × 240 mm, data matrix = 64 × 64, slice thickness = 4 mm, and 32 slices, 212 volumes).

### Surface-Based Structural Magnetic Resonance Imaging Analysis

FreeSurfer was used to analyze the structural MRI data. The details are described elsewhere (Dale et al., 1999), and it has been shown to be robust in exploring cortical and subcortical features (Rosas et al., 2002; Salat et al., 2004). Briefly, the primary preprocessing included Talairach transformation, removal of the non-brain tissue, and tissues segmentation. Three experienced researchers checked tissue segmentation. Subsequently, we obtained the subcortical volumes from the automated procedure implemented in FreeSurfer (Liu et al., 2016). The subcortical volumes (i.e., left thalamus, left hippocampus, left nucleus accumbens, left amygdala, right thalamus, right hippocampus, right nucleus accumbens, and right amygdala) were extracted and compared between the two groups. One-way analysis of covariance was performed to explore the subcortical volume differences after adding the effects of age, sex, and total brain size as nuisance variables (Davis et al., 2017; Reddan et al., 2018). Furthermore, correlations between gray matter volumes and depression scores were calculated by using Pearson’s analysis.

### Resting-State Functional Magnetic Resonance Imaging Data Preprocessing

Functional MRI data were processed and analyzed using FSL tools (FMRIB’s Software Library, version 6.00). Preprocessing steps included motion correction using MCFLIRT (Jenkinson et al., 2002), distortion correction with field map (FUGUE), and removal of non-brain structures using brain extraction tool (BET) (Smith et al., 2002), then the image was smoothed using a 5-mm FWHM Gaussian filter. The resulting functional MRI (fMRI) image was normalized to the structural image using a boundary-based registration (BBR) tool (Greve and Fischl, 2009). Then, the structural image was normalized to the MNI152-2mm template using linear and non-linear registrations (FLIRT and FNIRT) (Jenkinson et al., 2002).

The fMRI image was examined by researchers using a two-step quality checking procedure. First, data were excluded due to poor quality (movement >1 mm). Second, the registration quality is assessed by the amount of warp distortion according to the BBR-based function-to-structure realignment. Three subjects (two for MMD patients and one for healthy control groups) were not included in the analysis due to head movements during the fMRI scans.

### Resting-State Functional Connectivity Analysis

Because structural MRI analysis revealed smaller right amygdala volume in MMD patients than in healthy controls, we decided to select the right amygdala as the seed for the further functional MRI analyses. The seed of the right amygdala was defined from the Harvard Oxford subcortical structural atlas (90% threshold).

Resting-state functional MRI (rs-fMRI) analysis was conducted using a general linear model according to the well-documented procedures (Fox et al., 2006). By using brushes, we draw nuisance regions of interest of cerebrospinal fluid (CSF) and white matter (WM) in the standard MNI152 template layer by layer and then save them as a mask. The nuisance masks in the standard space are then converted to individual functional space, and the time series of CSF and WM were generated for each participant. Time signal extraction from the seed and nuisance regions was then performed for each subject. The mean time signal of each individual subject’s seed was set as an explanatory variable (EV) with realignment parameters, and the time series of nuisance regions were set as controlled variables. Significant differences in functional connectivity between MMD patients and healthy controls were determined using an independent sample *t*-test. The group-level statistical images were corrected using the parametric family-wise error method at the cluster level, cluster-forming threshold by Z > 2.3, and a corrected cluster significance threshold of *p* < 0.05.

### Statistical Analysis

Logistic regression analyses were used to explore the relationship between a dichotomized measure [remission (HAMD score ≤ 7) and treatment failure (HAMD score > 7)] assessed at both weeks 4 and 8 and potential factors [age, sex, right amygdala-nucleus accumbens (NAc) functional connectivity, and right amygdala volume] for calculating sensitivity, specificity, and the area under the receiver operating characteristic curve.

A bootstrapped mediation analysis was performed to examine the relationships among the initial right amygdala volume/right amygdala-based functional connectivity, Wechsler memory, and treatment efficacy (HAMD_*baseline*_—HAMD_4–*week*/8–*week*_). The PROCESS macro in SPSS was used with 5,000-bootstrap samples, which identified 95% confidence intervals (CIs) for model components. With the Wechsler memory as the mediator, we tested two models: (1) right amygdala volume as the independent variable and treatment outcome as the dependent variable and (2) coupling between right amygdala and NAc as the independent variable and treatment outcome as the dependent variable. These results determined the indirect effects of the Wechsler memory on right amygdala features and treatment outcome, yielding the 95% CIs of the indirect effects. A significant mediation occurred when the 95% CIs did not include zero (Hayes and Preacher, 2014).

## Results

### Demographic, Clinical Variables, and Wechsler Memory

**FIGURE 1:**
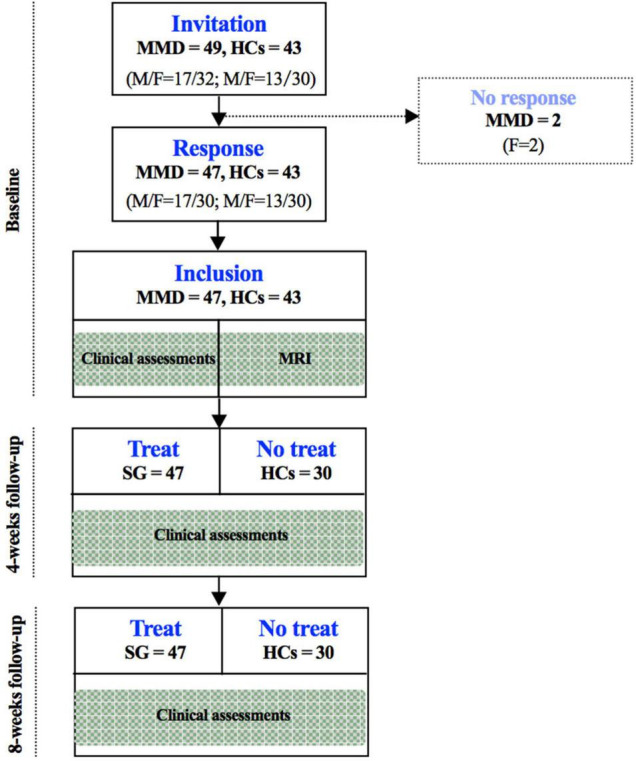
Flow chart of the participant enrollment and follow-up in this study. SG, Shugan Jieyu Capsule; HCs, healthy controls.

**FIGURE 2:**
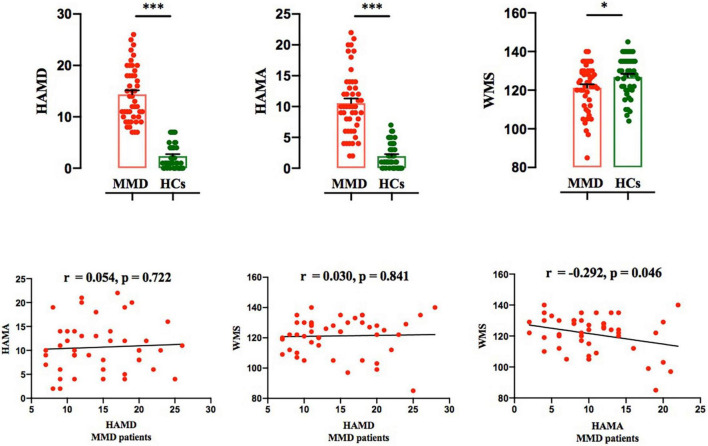
Comparison of HAMD and HAMA scores between the two groups, and correlation between HAMD and HAMA scores in MMD patients. Top panel*:* HAMD, HAMA and WMS scores were significantly larger in MMD patients than in HCs. Bonferroni corrected (*p < 0.05; ^***^p < 0.001). Bottom panel*:* Both HAMA and WMS scores were not significantly correlated with HAMD score in MMD patients; WMS score was significantly correlated with HAMA score in MMD patients. The error bars indicate standard error. HAMD, Hamilton Depression; HAMA, Hamilton Anxiety; WMS, Wechsler Memory Scale; MMD, mild-to-moderate depression; HCs, Healthy Controls.

The flow chart of the participant enrollment and follow-up in this study is summarized in Figure 1. Demographic (i.e., age, sex, and year of education) and clinical variables (HAMD and HAMA), as well as WMS in MMD patients and healthy controls (HCs), are presented in Table 1. The clinical variables, as quantified by HAMD (*t* = 13.245, *p* < 0.001) and HAMA (*t* = 10.151, *p* < 0.001), were significantly larger in MMD patients than HCs. In addition, neurocognitive characteristics, as measured by WMS (*t* = 2.332, *p* = 0.022), were significantly weaker in MMD patients than HCs (Table 1 and Figure 2, top panel). These suggest that MMD patients are not only accompanied by negative emotions but also with memory deficits.

**TABLE 1:** Participant demographics.

	MMD patients	Healthy Controls			
		
	Mean ± SD	Mean ± SD	df	χ^2^/*t*-value	*p*-value
Sex (male/female)	17/30	13/30	1	0.207	0.649
Age (years)	26.12 ± 5.06	26.27 ± 5.69	90	0.140	0.889
Age range (years)	18–35	22–34	-	-	-
Education (years)	19.75 ± 4.18	20.30 ± 3.97	90	-	-
Handedness (right/left)	47/0	43/0	-	-	-
HAMD	14.66 ± 5.60	2.37 ± 2.45	88	13.245	<0.001
HAMA	10.55 ± 5.18	1.97 ± 2.04	88	10.151	<0.001
WMS	121.58 ± 12.80	127.07 ± 10.39	88	2.332	0.022

*MMD, mild-to-moderate depression; HAMD, Hamilton Depression Rating Scale; HAMA, Hamilton Anxiety Rating Scale; WMS, Wechsler Memory Scale.*

### Correlations Between Clinical Variables and Wechsler Memory

For MMD patients, Person’s correlation results demonstrated that both HAMA and WMS scores were not significantly related to HAMD score (HAMA *vs*. HAMD: *r* = 0.054, *p* = 0.772; WMS *vs*. HAMD: *r* = 0.030, *p* = 0.841; Figure 2, bottom panel); Additionally, only HAMA and WMS scores yielded a significant result in MMD patients (*r* = −0.292, *p* = 0.046; Figure 2, bottom panel).

### Subcortical Gray Matter Volumes

**FIGURE 3:**
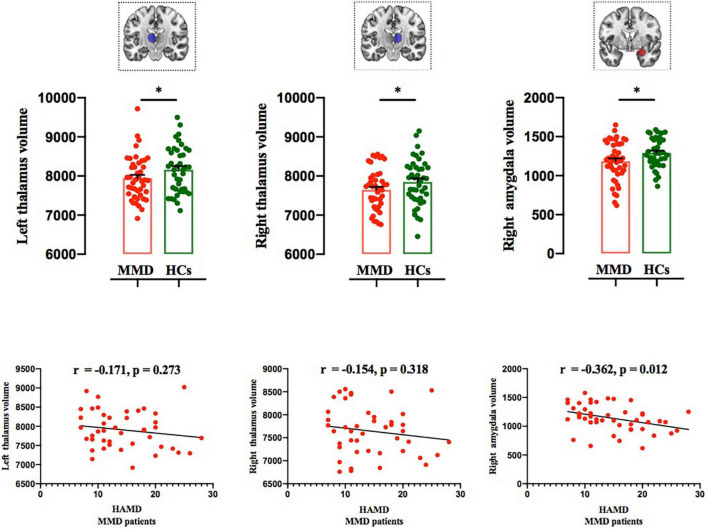
Comparison of subcortical volumes between the two groups, and correlations between subcortical volumes and HAMD scores in MMD patients. Top panel: The gray matter volumes of the left thalamus, right thalamus, and right amygdala were significantly smaller in MMD patients than in HCs (*p < 0.006; Bonferroni corrected). Bottom panel: Right amygdala volume was significantly negatively correlated with HAMD score, whereas the left and thalamus volumes were not related to HAMD score in MMD patients. MMD, mild-to-moderate depression; HCs, Healthy Controls.

Structural MRI results showed that the gray matter volume of the left thalamus, right thalamus, and right amygdala in MMD patients was significantly smaller than that of HCs after using variables of age, sex, education level, and total brain size as regressors (left thalamus: *F* = 8.000, *p* < 0.001; right thalamus: *F* = 8.912, *p* < 0.001; right amygdala: *F* = 5.728, *p* = 0.005; Table 2 and Figure 3; *p* < 0.006, corrected with Bonferroni correction).

**TABLE 2:** Differences in subcortical volumes between mild-to-moderate depression (MMD) patients and healthy controls (mean ± SD).

	Subcortical volume (mm^3^)				
Region	MMD patients	HCs	*F*	*p*	η_*p*_^2^
Thalamus-L	8,050.6 ± 678.3	8,090.2 ± 663.8	8.000	< 0.001*	0.218
Thalamus-R	7,746.2 ± 641.7	7,801.9 ± 654.3	8.912	< 0.001*	0.237
Hippocampus-L	3,940.4 ± 443.3	4,070.1 ± 418.2	2.785	0.046	0.089
Hippocampus-R	4,093.9 ± 475.1	4,233.4 ± 447.1	2.324	0.081	0.075
NAc-L	602.7 ± 91.2	589.9 ± 67.0	3.262	0.025	0.102
NAc-R	458.2 ± 65.8	478.1 ± 90.1	0.521	0.669	0.018
Amygdala-L	1,217.1 ± 192.9	1,226.1 ± 226.8	2.841	0.064	0.063
Amygdala-R	1,184.8 ± 249.5	1,292.4 ± 227.2	5.728	0.005*	0.115

NAc, nucleus accumbens; L, left; R, right; MMD, mild-to-moderate depression; *p < 0.006 (Bonferroni corrected).

The gray matter volume of the right amygdala was negatively correlated with HAMD score (*r* = −0.362, *p* = 0.012; Figure 3), whereas the gray matter volumes of the left and right thalamus were not correlated with HAMD score in MMD patients (*r* = −0.171, *p* = 0.273; *r* = −0.154, *p* = 0.318; Figure 3). No significant results were detected between the two groups in other subcortical and cortical volumes (left hippocampus: *F* = 2.785, *P* = 0.046; right hippocampus: *F* = 2.324, *P* = 0.081; left NAc: *F* = 3.262, *p* = 0.025; right NAc: *F* = 0.521, *p* = 0.669; left amygdala: *F* = 2.841, *p* = 0.064; Table 2; *p* < 0.006, corrected with Bonferroni correction).

### Seed-Based Functional Connectivity

**FIGURE 4:**
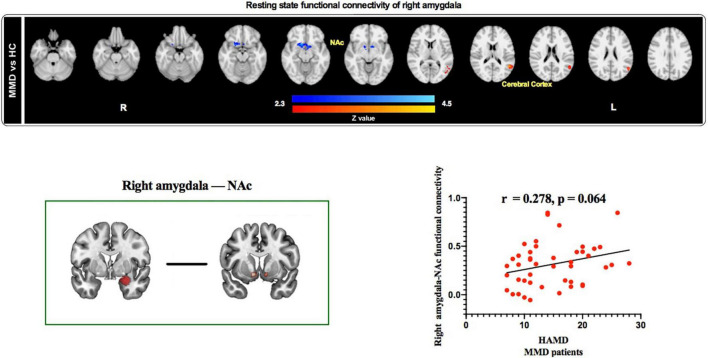
Resting state functional connectivity of right amygdala and correlation between right amygdala-based functional connectivity and HAMD scores in MMD patients. Top panel: The right amygdala exhibited weaker resting state functional connectivity with the NAc and stronger resting state functional connectivity with the cerebral cortex in MMD patients than in HCs. Bottom panel: Resting state functional connectivity between right amygdala and NAc was positively correlated with HAMD scores in MMD patients (marginal significant). NAc, Nucleus accumbens; HAMD, Hamilton Depression; MMD, mild-to-moderate depression; HCs, Healthy Controls.

Results from rs-fMRI indicated that the right amygdala showed significantly weaker functional connectivity with the NAc and stronger functional connectivity with the cerebral cortex in MMD patients than in HCs (*Z* > 2.3, *p* < 0.05 cluster-wise corrected; Table 3 and Figure 4, top panel). Interestingly, results identified a marginally significant positive relationship between HAMD score and right amygdala–NAc functional connectivity in MMD patients (Figure 4, bottom panel).

**TABLE 3:** Clusters that exhibited significant resting-state functional connectivity differences of right amygdala between mild-to-moderate depression (MMD) patients and healthy controls.

Area	Side	Brodmann’s area	MNI coordinates		
			Peak x/y/z	*Z*-value	Voxels
	**MMD patients >healthy controls**				
NAc	R	BA40	10 12 −12	4.04	289
	**MMD patients <healthy controls**				
Cerebral Cortex	L	BA31	−50 −54 16	4.05	408

*NAc, nucleus accumbens; L, left; R, right; MNI, Montreal Neurological Institute.*

### Longitudinal Investigation

Forty-seven patients from the original sample returned for follow-up evaluation after SG treatment at two time points (4 and 8 weeks), and the depression severity (assessed by HAMD) are presented in Table 4.

**TABLE 4:** Descriptive of Hamilton depression rating scale (HAMD) score for mild-to-moderate depression (MMD) patients and healthy controls during follow-up (mean ± SD).

	MMD patients	MMD patients	Healthy controls	Healthy controls
	
	4 weeks	8 weeks	4 weeks	8 weeks
HAMD	9.55 ± 5.36	5.95 ± 3.49	2.13 ± 2.34	2.16 ± 1.98

*MMD, mild-to-moderate depression; HCs, healthy controls; HAMD, Hamilton Depression Rating Scale.*

**FIGURE 5:**
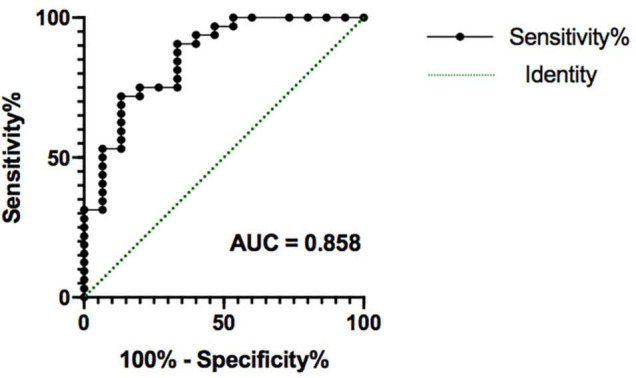
The receiver operating characteristic plot displaying sensitivity and specificity properties for a combined model formed by potential factors (age, sex, right amygdala volume, right amygdala-NAc functional connectivity). This model provided a good prediction of persistent depression obtained after 8 weeks of SG treatment, with area under the receiver operating characteristic curve (AUC) = 0.858 (*p* < 0.001). SG, Shugan Jieyu Capsule.

We used logistic regression to combine potential measures into a signal signature of outcome groups after weeks 4 and 8 of SG treatment separately. At 4 weeks, the model generated by the logistic regression analysis is not significant (*F* = 0.593, *p* = 0.988). At 8 weeks, the logistic regression analysis resulted in a significant model (*F* = 14.100, *p* = 0.007), showing that treatment outcome was significantly predicted by age (*t* = 4.358, *p* = 0.037) and right amygdala volume (*t* = 9.708, *p* = 0.002), whereas sex (*t* = 1.165, *p* = 0.280) and right amygdala–NAc functional connectivity (*t* = 1.165, *p* = 0.280) were not significant. This model provided a good prediction of developing depression after 8 weeks of SG treatment [area under the receiver operating characteristic curve = 0.858 (95% CI 73.6–98.1%); *p* < 0.001, Figure 5].

### Mediation Analysis

**FIGURE 6:**
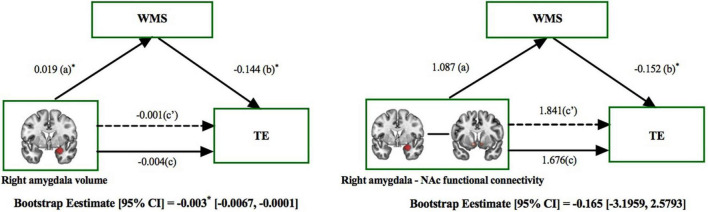
The mediating role of memory ability on the effect that right amygdala had on treatment efficacy. The mediation model included right amygdala features (subcortical volume and functional connectivity) as the independent variable, the effects on treatment efficacy (HAMD_*baseline*_–HAMD_4–wee*k/8–week*_) as the dependent variable, and the effects on memory ability [assessed by Wechsler Memory Scale (WMS)] as the mediator. Left panel: The effect of right amygdala volume on TE was mediated by the WMS. Right panel: The effect of right amygdala and NAc functional connectivity on TE was not mediated by the WMS. *p < 0.05; TE, Treatment Efficacy.

Mediation analysis revealed the indirect effect of right amygdala volume on treatment efficacy *via* Wechsler memory in patients with MMD [direct effect = −0.001, *p* > 0.05; indirect effect = −0.003, 95% CI (−0.0067, −0.0001), Figure 6, left panel]. This result preliminary provides evidence of Wechsler memory in mediating the indirect role of right amygdala reduction on treatment efficacy. In contrast, the effect of right amygdala and NAc functional connectivity on treatment efficacy was not mediated by the Wechsler memory in patients with MMD [direct effect = 1.841, *p* > 0.05; indirect effect = −0.165, 95% CI (−3.1959, 2.5793), Figure 6, right panel].

## Discussion

Amygdala is a key component of the emotional network, which has received extensive attention in a wide range of emotional processing, such as emotional perception, memory, and regulation (Kronenberg et al., 2009). In recent years, voxel-based gray matter morphology analysis and rs-fMRI show that amygdala volume abnormalities and dysfunction of several amygdala-related circuits are related to depression (Fox et al., 2005). Here, we adopted clinical assessments and MRI to illustrate the neural mechanisms before and after treatment in MMD. We found that MMD demonstrated structural and functional deficits in the right amygdala compared with HCs. Importantly, we also found that right amygdala volume at baseline served as a significant predictor to identify the treatment efficacy after 8 weeks of SG treatment for MMD, and especially, the effect of initially right amygdala volume on treatment efficacy was mediated by the memory ability. In conclusion, this work suggested that MMD was characterized by structural and functional deficits of the right amygdala, which could further influence the treatment effects of SG.

After 8 weeks of SG treatment, HAMD scores were significantly decreased in MMD (mean score < 7). For MMD, SG shows superior long-term efficacy. Lewis et al. (2012) showed that the improvement of early depressive symptoms might be an essential condition for successful treatment (Lewis et al., 2012). Our results show that the 8-week SG treatment has a good effect on MMD.

The findings of structural MRI showed that compared with HCs, the gray matter volume of the left thalamus, right thalamus, and right amygdala of MMD was significantly smaller. Importantly, the volume of the right amygdala of MMD was negatively correlated with the HAMD score. Consistent with the results of previous studies, this finding shows that depression is related to the anatomical changes of the right amygdala, and the degree of change is related to the symptoms of depression. Numerous studies have shown a decrease in amygdala volume in major depression; for instance, Hastings et al. (2004) demonstrated that after controlling the effect of total brain volume, the volume of the right amygdala in patients with depression decreased significantly compared with healthy female participants, which is consistent with the results reported in this study. The decrease in the volume of the right amygdala in MMD indicates that the amygdala in the brain development of patients with depression is abnormal, and the volume of the right amygdala decreases from the early stage of the disease (Hickie et al., 2007). Indeed, previous studies indicated that the size of the amygdala is negatively correlated with early depressive episodes, which is inferred that depression reduces the size of the amygdala (Kronenberg et al., 2009).

The rs-fMRI analysis showed that compared with HCs, the functional connectivity between the right amygdala and NAc in patients with MMD was significantly weaker, and the functional connectivity with the cerebral cortex was stronger. The amygdala is closely related to individual emotional processing, and it is also closely connected with other parts of the limbic system; in consequence, it was the core of neuroanatomical research on depression. Previous subgroup results from adults with major depressive disorder showed that the left amygdala has resting-state functional connectivity with prefrontal–limbic areas, whereas the right amygdala was mainly connected with the occipital lobe and subcortical regions (Tang et al., 2018). The NAc is widely associated with the prefrontal cortex and the limbic system (Tang et al., 2018). Especially, Green et al. (2019) found that the anhedonia is related to the deficits of the NAc. Decreased connectivity between the amygdala and NAc is associated with reward deficits in psychiatric patients. Interestingly, the functional connectivity between NAc and the right amygdala in MMD was positively correlated with depressive symptoms. Our results show that the connection pattern between the amygdala and NAc is related to anhedonia, and it can be regulated by depression, which is consistent with previous studies.

There is evidence that more severe depression may lead to a greater reliable degree of amygdala volume changes; thus, we related initial depression alteration in the right amygdala to long-term antidepressants outcome in well-defined MMD patients (Hamilton et al., 2008). The initial volume of the right amygdala emerged as a significant predictor to identify the efficacy of antidepressants in patients with MMD assessed after 8 weeks of SG treatment, whereas the coupling between right amygdala and NAc was not. These results highlight that SG may modulate brain structures rather than the functions to achieve therapeutic effects, which may strengthen the clinical implications targeting modulating specific targets to reduce depression.

There is an interesting result that mediation analyses showed the effect of right amygdala volume on treatment efficacy that was mediated by the Wechsler memory score in MMD patients. Previous studies have demonstrated that the mutual neural basis of depression and anxiety symptoms was closely related to cognitive control and emotional processing and was mediated by the functional connectivity between the amygdala and specific brain regions (including the dorsomedial prefrontal cortex, bilateral medial orbitofrontal cortex, left middle temporal gyrus, and lingual gyrus) (Spielberg et al., 2008; Andersen and Cui, 2009; Etkin et al., 2009). Indeed, the combination of the amygdala and these cognitive-related structures can make a detailed evaluation of emotional stimulus and conscious awareness; thus, the impaired memory performance may be significantly correlated with both limbic regions and negative emotional information (Canu et al., 2015). In addition, the involvement of emotional circuitry and cognitive control in patients with depression represents the complexity of psychopathology caused by the interaction between anxiety and depression, all of which may occur at the neural network level. Noteworthy, there are research findings that gene polymorphisms involved in serotonin biosynthesis, transport or signal transduction, as well as neurotrophins factors such as brain-derived neurotrophic factor (BDNF), are associated with an increased risk of depression, which may lead to structure and function differences of the amygdala (Munafo et al., 2008; Roiser et al., 2009; Scharinger et al., 2010). The products of BDNF may promote neurogenesis and protect against excitotoxicity in both the hippocampus (Khaspekov et al., 2004; Scharfman et al., 2005) and striatum (Perez-Navarro et al., 1999; Mohapel et al., 2005) and mediates the increase of amygdala volume in samples of patients with drug-induced depression. Antidepressants can increase the expression of BDNF in the hippocampus of rodents (Nibuya et al., 1995) and humans (Chen et al., 2001) and that BDNF and its receptor tyrosine kinase B are involved in amygdala-dependent learning (Rattiner et al., 2004; Chhatwal et al., 2006). Our data suggest that amygdala volume affected antidepressant efficiency indirectly through memory performances, which implies that memory ability plays an intermediate role in the relationship between amygdala morphology and depression.

### Limitations and Outlook

It is worth noting that there are some limitations worth considering in this study. First, the relatively small sample size limited the exploration of the relationship between subcortical dysfunctions and symptoms in MMD patients. Second, further studies are needed to investigate the extent to which our findings are specific to MMD to further explore commonalities and differences between psychiatric disorders linked to amygdala morphology. Third, we only focus on the effect of SG treatment in this study; there is no placebo, which prevents us from exploring the effect and mechanism of placebo. Although this study has limitations, it also raises further suggestions of initially right amygdala volume served as a significant predictor to identify the treatment efficacy after 8 weeks of SG treatment for MMD patients, and especially, the effect of initially right amygdala volume on treatment efficacy was mediated by the Wechsler memory. In the future, these results could be replicated and summarized in psychiatric disorders by using longitudinal studies or large sample size studies. A more detailed understanding of the anatomy of the amygdala underlying depression in MMD will help to formulate more targeted treatment protocols.

## Data Availability Statement

The raw data supporting the conclusions of this article will be made available by the authors, without undue reservation.

## Ethics Statement

The studies involving human participants were reviewed and approved by the Ethics Committee of Shanxi Medical University. The patients/participants provided their written informed consent to participate in this study.

## Author Contributions

HL: data curation, formal analysis, and writing–original draft. JW: data curation and writing–review and editing. SL and ZL: investigation and resources. YX: supervision, funding acquisition, and writing–review and editing. All authors contributed to the article and have approved the final manuscript.

## Conflict of Interest

The authors declare that the research was conducted in the absence of any commercial or financial relationships that could be construed as a potential conflict of interest.

## Publisher’s Note

All claims expressed in this article are solely those of the authors and do not necessarily represent those of their affiliated organizations, or those of the publisher, the editors and the reviewers. Any product that may be evaluated in this article, or claim that may be made by its manufacturer, is not guaranteed or endorsed by the publisher.
